# Examining Whistleblowing Intention: The Influence of Rationalization on Wrongdoing and Threat of Retaliation

**DOI:** 10.3390/ijerph19031752

**Published:** 2022-02-03

**Authors:** Jawad Khan, Imran Saeed, Muhammad Zada, Amna Ali, Nicolás Contreras-Barraza, Guido Salazar-Sepúlveda, Alejandro Vega-Muñoz

**Affiliations:** 1 Department of Business Administration, Iqra National University, Peshawar 25100, Pakistan; jawadmarwat1@gmail.com (J.K.); Am_pk97@yahoo.com (A.A.); 2 Institute of Business and Management Sciences, The University of Agriculture, Peshawar 25130, Pakistan; imranktk1984@gmail.com; 3 Business School, Henan University, Kaifeng 475000, China; 4 Facultad de Economía y Negocios, Universidad Andres Bello, Viña del Mar 2531015, Chile; nicolas.contreras@unab.cl; 5 Departamento de Ingeniería Industrial, Facultad de Ingeniería, Universidad Católica de la Santísima Concepción, Concepción 4090541, Chile; gsalazar@ucsc.cl; 6 Public Policy Observatory, Universidad Autónoma de Chile, Santiago 7500912, Chile

**Keywords:** perceived seriousness of wrongdoing, workplace, perceived threat of retaliation, rationalization, whistleblowing intentions, anticipated regret

## Abstract

Whistleblowers who expose wrongdoing often face several concerns, pressures, and threats of retaliation before reaching a final decision. Specifically, this study examines the effects of perceived seriousness of wrongdoing (PSW) and perceived threat of retaliation (PTR), as well as the impact of rationalization (RNL), comparing perceived seriousness of wrongdoing, perceived threat of retaliation and whistleblowing intention. Furthermore, this study aims to determine the mediating effect of anticipated regret (AR) on the relationship between perceived seriousness of wrongdoing and whistleblowing intention. We validated our model by analyzing data gathered across three stages from employees in the telecom sector in Pakistan. The key findings of our research may be summarized as follows: (i) individuals’ willingness to ‘blow the whistle’ increases as a result of perceived seriousness of wrongdoing; (ii) whistleblowers are more likely to opt to remain silent if they anticipate a greater threat of retaliation, and (iii) our study establishes a positive connection between perceived seriousness of wrongdoing and whistleblowing intention, indicating that perceived seriousness of wrongdoing enhances people’s willingness to blow the whistle, and whistleblowers are more likely to choose to emerge if the behaviour is more serious in nature; (iv) the data we have uncovered indicates a moderating role of rationalization in regulating the connections between perceived seriousness of wrongdoing, perceived threat of retaliation, and whistleblowing intention; and (v) the findings demonstrate that anticipated regret mediates the connection between perceived seriousness of wrongdoing and the intention to report wrongdoing. Additionally, the results are discussed in terms of their significance for corporate ethics researchers and managers, as well as for end-users who are interested in whistleblowing.

## 1. Introduction

In July 2010, Congress approved the Dodd-Frank Act, which established the US Securities and Exchange Commission (SEC) Whistleblower Initiative in response to the global financial crisis of 2008. For over a decade, the program has produced high-quality recommendations that have helped the SEC to uncover and stop fraud schemes while protecting investors. Approximately $700 million in compensation for whistleblowers has been given out by the SEC Office of the Whistleblower since 2012. Penalties against wrongdoers resulting from SEC efforts relating to the disclosures totaled more than $2.7 billion, which was paid out in the form of fines and penalties. According to the American Council on Fraud and Ethics’s 2020 Report to the Nations, there has been a considerable increase in the number of complaints received via business hotlines or reporting systems since 2010. Additionally, individuals should consider the SEC Whistleblower Program as another essential weapon in their collection of tools for exposing fraud and wrongdoing. Whistleblowers who met the criteria for the ACFE’s award received around $368 million in compensation, including the five highest awards in the program’s history—two awards of over $50 million and one each of $39 and $37 million. In 2020, hard work took place to process claims faster than ever before, even with the impact of COVID-19. This has allowed the distribution additional rewards [1]. The SEC Whistleblower Program is effective in uncovering fraud for five key reasons.

1. Strong financial incentives for whistleblowers
2. Anonymous reporting
3. Most individuals are eligible to receive awards
4. SEC rejects “gag clauses”
5. Protections for SEC whistleblowers

The Association of Certified Fraud Examiners (ACFE), the world’s premier anti-fraud organization, published a report on professional fraud and abuse for the tenth time in 2018, marking the organization’s establishment as a global leader in the field of fraud prevention. According to the ACFE’s most recent study, which examined 2690 real cases from 125 countries, whistleblowers were responsible for detecting or exposing 40% of fraud situations [2]. According to the ACFE, workers are most likely to blow the whistle (21%), followed by customers (14%), unidentified individuals (14%), suppliers (8%), outsiders (5%), rivals (3%), and owners of the organization (2%). This study has highlighted issues regarding why someone who witnesses misconduct takes the option to either blow the whistle or keep quiet [3,4,5,6,7].

Whistleblowing is a vital tool to prevent and uncover corporate misconduct, whether in the public or private sector [8,9,10]. To begin with, when the notion of whistleblowing was initially introduced in the 1970s, it was widely recognised across a broad variety of disciplines, especially those in the humanities such as sociology and psychology. It has since gained widespread acceptance and has also achieved widespread acceptance among the manufacturing industries [11,12]. Several significant studies on whistleblowing were published in the 1980s, including one by [13] that is still in use today, which defined whistleblowing as “a process involving at least four elements: (i) the whistleblower: a former or current member of an organization who is aware of a wrongdoing, but generally lacks the authority or power to make the required changes; (ii) the whistleblowing act: the act of disclosing an illegal, immoral, illegitimate practice to persons or organizations that might be able to bring about change; (iii) the complaint receiver: a third party (external whistleblowing) or someone other than or in addition to the immediate supervisor (internal whistleblowing); and (iv) the organization: a public or private organization who is the target of the whistleblowing and who will be called upon to respond (or not) to the disclosure of wrongdoing.” It is argued in [13] that, when it comes to reporting whistleblowing, it should not be seen as an act of employee disobedience. When someone comes out to report misbehavior, they are in reality opposing the power structure of the institution in which they work. To be clear, a whistleblower is not a criminal; rather, he or she is a person who feels passionately enough about an injustice to come forward and share information that might help the organisation and increase public safety [14].

**Figure 1:**
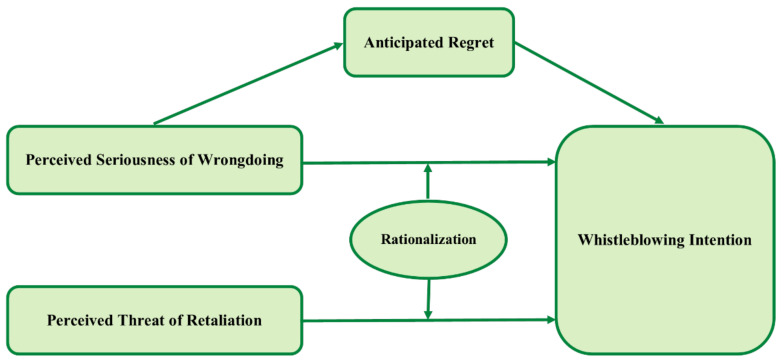
Theoretical Framework.

Culiberg and Mihelič [15] stated that a whistleblower must consider several factors before deciding whether to come forward with information about wrongdoing. Several factors, including the powerful position of wrongdoers, lack of support, and a fear of loss with respect to the organization, have been identified as contributing to whistleblowers’ tendency to keep quiet when injustice is seen [6,16,17,18]. However, the perceived threat of retaliation is a component that has yet to be thoroughly examined [11,19]. Perceived threat of retaliation (PTR) is a whistleblower’s estimate of the genuine amount of threat they may face as a consequence of disclosing wrongdoing inside an organization. Employees may be less likely to come forward with information that might result in retaliation due to such threats, which include the potential of being fired, being treated unfairly, or encountering intimidation or harassment from coworkers [20,21,22]. An individual will be less inclined to come forward and report wrongdoings because of the severity of retaliation. In certain cases, a boss may threaten to dismiss employees who know about wrongdoing inside the organization. However, are such threats genuine or merely a bluff? Rationalization (RNL) is necessary to determine the threat level. It is possible to characterize the role of rationalization in whistleblowing as a cognitive reasoning process that drives the decision to come forward [5,23,24]. To judge their actions (or inaction) against their standards of morality, whistleblowers must go through this process [8,22,25,26]. Another factor to consider is why an observer decides to make a report of wrongdoing: what inspires an observer to take action to help others? Previous research has shown that various predictor variables influence people’s intention to report wrongdoing when they see it. These variables include individual and situational factors and the environment (e.g., workplace spirituality, organizational commitment, monetary rewards, and organizational support) [22,27,28,29,30,31,32,33,34]. However, as indicated by [19,35,36], there are contradictory findings on the relationships between these elements, necessitating further investigation. Previous studies have reported conflicting results regarding the influence of perceived seriousness of wrongdoing on whistleblowing intention. Researchers have shown that the presence of perceived seriousness of wrongdoing increases the likelihood of someone making a whistleblowing initiative [28,29,37,38,39], but some researchers claim that this has had no impact [15,27,30,40,41]. Perceived seriousness of wrongdoing is described as a person’s assessment of the extent of the repercussions of unlawful, immoral, or illegitimate behavior [6,10,27,40]. A study conducted by [35] stated that, as a result of the poor link between perceived seriousness of wrongdoing and whistleblowing intention, there is certainly a need for a moderator variable. Perceived seriousness of wrongdoing, like perceived threat of retaliation, requires an RNL approach in order to evaluate the impact of the wrongdoing before the whistleblower reports. Using the context above as motivation, the purpose of this study is to look at the relationship between perceived threat of retaliation, perceived seriousness of wrongdoing, anticipated regret, and its impact on whistleblowing intention, as moderated by the rationalization approach. We validated our model by using employees in Islamabad’s telecom industry. We selected this group because the employees of these organizations have the maximum chance to encounter a wide range of wrongdoing throughout their employment [42]. Furthermore, similar employees have already participated in earlier studies on whistleblowing [15,40,42,43,44]. Moreover, research on whistling in developing nations, particularly in South Asia, is extremely uncommon [20,21]. While there have been countless studies on whistleblowing in various parts of the globe [45,46], most have been undertaken in developed countries, and there is still a shortage of knowledge from emerging countries, such as Pakistan. In terms of occupational fraud and abuse, the Southern Asia area has the highest score, with Pakistan coming in as second worst, along with nations such as India, Afghanistan, Bangladesh, and Maldives (as indicated above) [2]. There is an urgent need for a study, particularly in Pakistan, because of the lack of evidence and the large number of fraud cases the ACFE has identified. Our research adds to the body of knowledge on whistleblowing. Whistleblowing is flavor of the day as stated by [19,20], but needs further study [19]. There is a lack of scholarly research on retaliation towards whistleblowers; therefore, the threats faced by whistleblowers are not well studied. Along the same lines, ref. [47] investigating the implications of whistleblowing might assist both present and prospective whistleblowers in avoiding retaliation by providing them with the knowledge they need. Regarding perceived seriousness of wrongdoing and whistleblower intention, we examined whether anticipating regret acts as a mediator. Second, by including RNL as a moderator in the link between the two primary components, this research improves the notion of the ‘whistleblowing triangle’ [24]. Third, our research contributes to the body of knowledge by providing evidence in the context of developing countries [27,34,41,48,49], such as Pakistan. Whistleblowing research has been conducted in a few South Asian nations; however, in Pakistan, studies on this topic are uncommon. This paper is divided into three sections: theoretical background, hypothesis development, and research methodology, in which data analysis is provided. Finally, we evaluate our findings and provide suggestions that may be beneficial to academics and practitioners. The research structure shown in Figure 1 was used for testing.

## 2. Theoretical Background and Development of Hypotheses

### 2.1. Theory of Planned Behavior (TPB)

According to [50,51], the theory of planned behavior (TPB) has previously been shown to be an effective theoretical tool for predicting and studying ethical or morally wrong conduct [38,52,53,54]. Ajzen studied TPB as a useful conceptual model for evaluating the desire to engage in immoral activities. Predicting an individual’s intention to perform a certain action at a specific time and location is the goal of the Theory of Planned Behavior (TPB). People’s self-control is central to the theory’s application, which aims to include all human conduct. This model relies heavily on the concept of behavioural intent, which is shaped by one’s beliefs about the probability that one’s actions will produce the desired results and one’s assessment of the associated risks and advantages. The TPB seems to be especially well-suited to describing whistleblower intentions, which are actions motivated by a very complicated psychological set of factors [30,55,56]. Ajzen’s theory is also widely recognized as a useful mechanism for analyzing variances in attitudes and intentions, and differences in intention and behavior across individuals. It is hoped that by trying to explain whistleblowing using TPB, some of the constraints of previous studies would be overcome and better understanding reached of the often-observed distinction between attitude and behavior. According to the TPB, the intention to engage in an activity is a result of three types of underlying belief that are conceptually distinct from one another: “(1) attitude toward the behavior, which is determined by beliefs about the consequences of that behavior; (2) subjective norms, determined by normative beliefs; and (3) perceived behavioral control, which is determined by beliefs about the resources and opportunities available to perform the behavior [50,51]”. A person’s attitude defines how much they accept or disapprove of a certain action or situation. In general, a person’s attitudes are developed because of their perceptions of their behavior and the consequences of that behavior. TPB posits that one’s views about a particular action’s effects help shape one’s attitude toward that action. A person’s level of belief and the value placed on particular outcomes affect their attitude toward an action. The employee’s attitude toward whistleblowing is the sum of their perceptions about the consequences of whistleblowing and their subjective assessment of those consequences (the degree to which a person has a positive or negative evaluation of whistleblowing). The outcomes of whistleblowing include avoidance of harm to an organization, control of corruption, improvement of public interest, an employee’s completion of their obligation, and moral pleasure, as stated in the goals of whistleblower protection laws [37]. Whistleblowing is often seen as a positive workplace activity that should be promoted.

### 2.2. Perceived Seriousness of Wrongdoing and Whistleblowing Intention

Whistleblowing is defined as “the disclosure by organizational members (former or current) of illegal, immoral, or illegitimate practices under the control of their employers, to persons or organizations that may be able to effect action” [13]. The degree to which unlawful, unethical, or illegitimate conduct may damage whistleblowers is known as the seriousness of the wrongdoing.

The whistleblower first evaluates the depth of the wrongdoing before whistleblowing. This activity is performed to judge the seriousness and course of action to be completed before it causes harm to the organization or employee [6,29,38,57,58]. With respect to perceived seriousness of wrongdoing, [36] states that an increase in the seriousness of wrongdoing will likely stimulate and impact a whistleblower’s intent to come forward with their information. Furthermore, the magnitude of wrongdoing negatively impacts stakeholders. As a result, the whistleblower decides to take action simultaneously [26,40]. As previously stated, this illustrates that, when serious wrongdoings occur frequently, it increases the likelihood that whistleblowers will own up to and disclose wrongdoings on their own [22,27,40]. When the wrongdoing is substantial, a whistleblower is more likely to take action [58]. In addition, a wrongdoing’s seriousness is linked to the intensity of inescapable repercussions, and this influences people’s ethical decision-making [30]. The perceived seriousness of wrongdoing may also be related to the accounting notion of “materiality,” which is specified in financial statements by professional accountants as “a concept that defines why and how specific issues are essential for a company or a business sector to highlight,” and which may also have implications for some considerations. Like perceived seriousness of wrongdoing, it is necessary for an employee to call attention to wrongdoing [59,60]. According to prior research, perceived seriousness of wrongdoing seems to be associated with an increased likelihood of blowing the whistle [17,28,61,62]. According to preliminary data, the seriousness of wrongdoing witnessed in organizations has a favorable impact on the whistle-blowing process; that is, the whistleblower will report the perceived seriousness of wrongdoing if the wrongdoing is serious enough [28].

**Hypothesis** **1** **(H1).** *The perceived seriousness of wrongdoing has a positive effect on whistleblowing intention.*

### 2.3. Rationalization as a Moderator between PSW and Whistleblowing Intention

According to researchers, perceived seriousness of wrongdoing is a significant positive predictor of whistleblowing intention [28,63,64,65]. It is difficult to pin down the perceived seriousness of wrongdoing approach. As a result, perceived seriousness of wrongdoing might vary based on the whistleblower’s intentions and depending on the cultural setting. It is possible that seriousness does not have enough of an impact on a person’s whistleblowing intentions because of the effect of factors such as group norms and organizational culture on the severity of wrongdoing [63,66]. A new study shows that perceived seriousness of wrongdoing is strongly associated with public accountants’ desire to report wrongdoing in Barbados [40]. When weighing the pros and cons of blowing the whistle, one factor to consider is the seriousness of the wrongdoing. Most people do nothing until they are convinced that the wrongdoing behavior is alarming [35]. Rationalization must be considered in this situation. Whistleblowing intent tends to rise when there is a considerable risk of harm [30], and this sense of threat is a cognitive part of the rationalization. It is stated in [45] stated that the rationalization approach facilitates the whistleblower in realizing the significance of the harm and threats regarding the wrongdoing they have observed [21].

**Hypothesis** **2** **(H2).** *Rationalization moderates the relationship between perceived seriousness of wrongdoing and whistleblowing intention.*

### 2.4. Perceived Threat of Retaliation and Whistleblowing Intention

Whistleblowers face several threats that have negative implications and even mentioning wrongdoing might result in serious repercussions. There are several threats related to blowing the whistle that influence whether someone will go forward with their information (such as those from wrongdoers, organisations, or third parties) [21,22,43]. Retaliation threats are prevalent, according to past research. However, not all whistleblowers face retaliation [36]. The seriousness of threats varies depending on pressure from employees, the organization, line managers, and also, in some situations, workplace bullying, verbal harassment, and even loss of the whistleblower’s reputation [6,29]. Not all threats should be taken seriously; based on the perceived seriousness of threat and the whistleblower’s psychological well-being, they will determine whether or not they should blow the whistle [32,67]. Threats may make a whistleblower reconsider disclosing wrongdoing, preventing whistleblowing from accomplishing its job. When a whistleblower perceives a threat, it causes worry, fear, and low self-esteem, all of which influence their choice [68]. According to the power theory [69], the degree of threat is often inversely proportional to the amount of power wielded by the whistleblower. As a consequence, when the observer is more dependent on the organisation than the offender, the threat increases [17]. Thus, potential whistleblowers are more concerned about the serious threats they may face if they come out with information. In a meta-analysis [35] utilizing data from a sample of 21 researchers, it was shown that the threat of retaliation is adversely connected with the intention to come out with information. Furthermore, prior studies by [31], refs. [68,70,71] found that the possibility of retaliation reduces the chance of people saying they will blow the whistle when asked. Another study by [7] perceived that threat of retaliation lowers when effective signals regarding whistleblowing are made accessible.

**Hypothesis** **3** **(H3).** *Perceived threat of retaliation has a negative effect on whistleblowing intention.*

### 2.5. Rationalization as a Moderater between Perceived Threat of Retaliation and Whistleblowing Intention

The threat level in various situations of whistleblowing has been observed in previous research, at varied percentages. For example, [14] demonstrates that retaliation is widespread in three nations’ public sectors (Australia, Norway, and the United States), with a frequency ranging from 4–22%. A study conducted by [32] stated that, when it comes to the well-being of whistleblowers in South Korea, researchers found that retaliation has a substantial influence on the health of people who disclose wrongdoing. Ref. [72] stated that, when observers notice unlawful, immoral, or unethical acts and feel a significant threat level, they should go through a rationalization approach before determining whether or not to report. This is a psychological approach that allows observers to identify the difference between two scenarios, for example, between what really transpired and what should have occurred in a given situation [5]. The ‘whistleblowing triangle’, according to this notion, illustrates how rationalization may be utilized as a cognitive rationale for reporting wrongdoing or leaking confidential information. As a result of rationalization, observers might reinterpret their report of wrongdoing [24]. For example, whistleblowers may assume that (a) since they are protected by the law, the threats they confront are insignificant; (b) if they believe wrongdoing has been committed, they have the option of confessing this in private; or (c) they would have the support of their coworkers and superiors. Prior research by [22] shows that the intention to blow the whistle is affected by rationalization. Observers must decide how they will speak out after opting to report misconduct, which may be done via internal, external, or via other confidential means. Observers have no clear pattern for making decisions on how to report wrongdoings. Scholars assert that the perceived importance of the wrongdoing may influence this decision, although the argument goes on [15].

**Hypothesis** **4a** **(H4a).** *Rationalization moderates the relationship between the perceived threat of retaliation and whistleblowing intention.*

### 2.6. Perceived Seriousness of Wrongdoing, Anticipated Regret and Whistleblowing Intention

The seriousness of the wrongdoing has an influence on the possibility of having regrets about not reporting it sooner rather than later. According to the definition, the seriousness of wrongdoing relates to the extent to which a certain unlawful behaviour may cause harm to others after they show an intention to blow the whistle [55,73]. In the case of more serious wrongdoings, it would be assumed that the potential for more public damage existed, resulting in a greater anticipated regret for staying quiet in the presence of such wrongdoings. According to previous research, potential whistleblowers are motivated to come forward when there is significant wrongdoing, and they feel a personal obligation to reveal the wrongdoing [32,74]. Previous research has shown that most people are afraid of regretting their decisions; thus, when faced with a choice between multiple different courses of action, they will choose the one that would cause them the least amount of regret [75]. Potential whistleblowers must weigh the advantages of blowing the whistle against the hazards of remaining silent, and the likelihood of regret may influence their choice. In light of the anticipated regret associated with being quiet, it is feasible that people will be more ready to participate in whistleblowing [70]. This line of reasoning is compatible with conceptual ideas addressing the link between anticipated regret and the intention to blow the whistle [76]. Prospective whistleblowers are more likely to blow the whistle if they are feeling significant anticipated regret about remaining silent.

**Hypothesis** **4b** **(H4b).** *Anticipated regret mediates the relationship between perceived seriousness of wrongdoing and Whistleblowing Intention.*

### 2.7. Control Variables

Gender differences in whistleblower behaviour have been reported in the past [77]. When deciding whether to blow the whistle, men and women may respond differently. In males, a strong sense of self-efficacy and self-esteem may lead to whistleblowing, but in females a strong sense of obligation to the public good may lead to whistleblowing. Males are more likely than females to use external whistleblower channels, according to a previous study. Another study found that women were more likely than men to report an incident anonymously [57,78]. It has also been found that work experience and the age of the whistleblower have an impact on their intentions. Working for a company for a longer duration increases the possibility of whistleblowing intentions because employees with more years of experience think they have more power and are more credible as whistleblowers [70,79]. According to prior research, age may also have an influence on whistleblower intentions. More data on the influence of age and employment experience on whistleblowing intentions has been provided by [71,80].

## 3. Research Methodology

### 3.1. Sample and Data Collection

The data were collected from 450 employees of telecommunications companies in Islamabad, Pakistan, through emails and also through physical visits to office premises, by adopting a convenience sampling technique at the start of 2020. Several procedural techniques were investigated in this study to reduce the common method variance [81], e.g., by collecting data at different time intervals with a gap of one month (Time 1, Time 2, and Time 3). A unique ID was given to each participents to confirm their particiation at all three waves. There would be no disclosure of any information gathered via the questionnaire. Participants were informed of the study’s purpose and confidentiality prior to completing and returning the questionnaire, which was sent via email and in a sealed envelope with instructions on how to complete and return it. After completion, the survey was returned by respondents in a sealed envelope from those who had filled in the questionnaire with paper and pencil, and also we received other questionnaires through email addresses. At Time 1, data were collected regarding perceived seriousness of wrongdoing and PTR, and 439 responses were received (97.55%). At Time 2, data were collected regarding anticipated regret and RNL; at this stage, 386 responses were received (87.92%). Data were collected regarding whistleblowing intention at Time 3, and 366 responses were received (94.81%). After scrutinizing the final data, 12 questionnaires were excluded due to missing data. The final sample size was 354. This sample consisted of 221 (62.78%) men and 133 (37.21%) women (Table 1). The respondents’ age was between 30–34 years, and their tenure with the organization was 1–7 years, with maximum participation. Of all respondent, 4.8% were HSSC (Higher Secondary School Certificate) and 18.5% had bachelor’s qualifications. Masters and MS/Phil accounted for 65.3% and 11.6%, respectively. The number of responses collected might be adequate to establish early conclusions regarding possible links by comparing comparable research with a sample size of 352 [20].

### 3.2. Measure

All aspects of this study were measured using a 5-point Likert scale, ranging from 1 = “strongly disagrre” to 5 = “strongly agree”.

#### 3.2.1. Whistleblowing Intention

To measure whistleblowing intentions, the scales in [82] have been used. Whistleblowing intentions have been measured using four items (Internal Whistleblowing), which require the respondent to state whether they would report the questionable acts.

#### 3.2.2. Perceived Seriousness of Wrongdoing (PSW)

To measure PSW, a scale developed by [83] was used. Respondents were asked whether during the previous 12 months they had personally observed or obtained direct evidence of any of 14 activities.

#### 3.2.3. Perceived Threat of Retaliation (PTR)

To measure PTR, a five-item scale adopted from the study of [84] were used.

#### 3.2.4. Rationalization (RNL)

To measure RNL, we adapted the five-item scale developed by [23,25,85], with modifcations.

#### 3.2.5. Anticipated Regret

Anticipated regret was assessed with a seven-item scale, developed by [86].

## 4. Results

### 4.1. Confirmatory Factor Analysis

To analyse the factor structure of our survey measures, we utilised IBM SPSS Amos 22 to run a series of confirmatory factor analyses [87,88]. In order to achieve the estimate criterion of the required parameters to sample size ratio, we included studied variables in the tests (1:5; [89]. Perceived seriousness of wrongdoing, perceived threat of retaliation, anticipated regret, rationalization, and whistleblowing intention were the first five factors we investigated. The data was reasonably well matched by the proposed five-factor model: χ^2^/df = 2.03, *p* < 0.001; comparative fit index (CFI) = 0.91; root mean square error of approximation (RMSEA) = 0.03; GFI = 0.89; root mean square residual (RMR) = 0.03. Table 2 shows the results of a series of model comparisons. We moved on to our major studies, since we were able to uncover support for our proposed factor structure.

### 4.2. Common Method Variance (CMV) and Variance-Inflation Factor (VIF)

On the recommendation of [90], a single-factor test proposed by Harman was used to check for problems associated with common method variation. There may be biases associated with a responder, a researcher, or the instrument that was used to collect the data. Only 28.74% of the variance was attributed to a single component, which is far lower than the 50% threshold [91], so we conclude that no threat of common method bias exists. Regarding possible multicollinearity problems [92], the variance-inflation factor (VIF) values of variables were examined. The results showed that the internal VIF values ranged from 1.142 to 1.312, well below 2 [93]. Therefore, no major multi-linearity problems exist, as shown in Table 3.

### 4.3. Descriptive Statistics and Correlations

The means, standard deviations, and correlation coefficients for all variables in this research are listed in Table 3. Perceived seriousness of wrongdoing has a positive correlation with whistleblowing intention (r = 0.757 **, *p* 0.01), and PSW has a significant correlation with whistleblowing intention (r = 0.470 **, *p* 0.01). Additionally, the more significant correlation is between rationalization and anticipated regret (r = 0.877 **, *p* 0.01); (see Table 3 for additional data). These findings provide preliminary significant evidence for our hypothesis.

### 4.4. Hypothesis Testing

A hierarchical regression analysis was performed to determine whether perceived seriousness of wrongdoing and perceived threat of retaliation led to whistleblowing intentions. As seen in Table 4, a statistically significant link existed between perceived seriousness of wrongdoing and whistleblowing intention (β = 1.066, *p* < 0.001), which supports Hypothesis 1 (Model 3). As for Model 4, perceived threat of retaliation negatively affects perceived seriousness of wrongdoing (β = −0.509, *p* < 0.001), thus supporting Hypothesis 3. As displayed in Table 4, PSW was significantly related to anticipated regret (β = 0.220, *p* < 0.001, Model 1), and anticipated regret had a significant influence on whistleblowing intention (β = 0.013, *p* < 0.01, Model 5). To further confirm the mediation effect of anticipated regret (perceived seriousness of wrongdoing → anticipated regret → whistleblowing intention), we adapted bootstrapping of 5000 cases’ analyses with a 95% bias-corrected confidence interval. We found that anticipated regret partially mediated the path between perceived seriousness of wrongdoing and whistleblowing intention (β = −0.07, −0.1253 to −0.0268), and that the indirect effect exists as a consequence of the confidence interval excluding zero between the upper and lower boundaries. (see Table 5). After analyzing the mediator variable, the values in Table 5 reveal and confirm the mediating role, thus confirming Hypothesis 4b.

### 4.5. Interaction Effect

**Figure 2:**
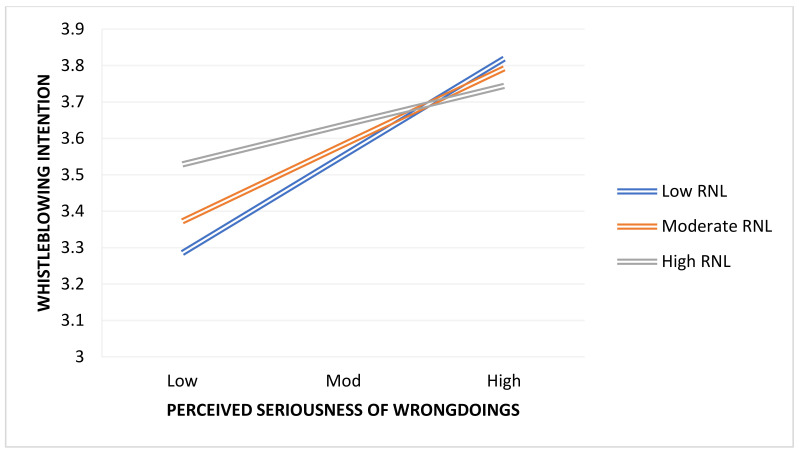
PSW*RNL = Whistleblowing Intention.

**Figure 3:**
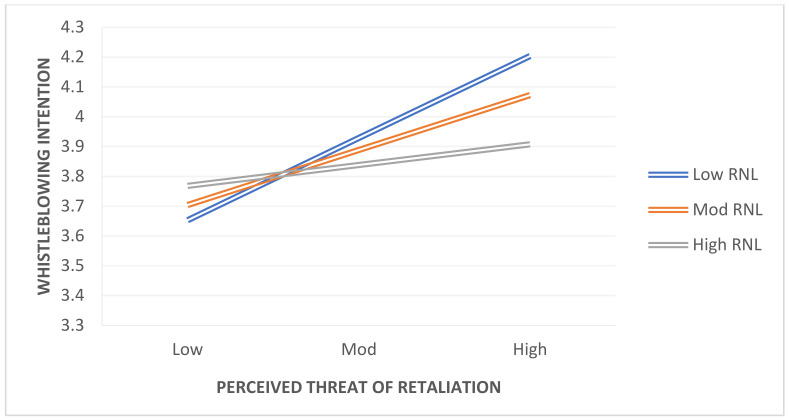
PTR*RNL = Whistleblowing Intention.

By examining the link between perceived seriousness of wrongdoing and perceived threat of retaliation, we determined the influence of rationalization on whistleblowing intention. To quantify the interaction effects, we used the methods developed by Hayes (2018). To begin, we evaluated the model without taking into account the interaction effects. Following that, we retested the model by including interaction effects and determining the degree of significance. Finally, we used the Hayes PROCESS macro to create a visual graph. Table 6 and Table 7 show the findings for interaction effects, and we obtain the predicted results, with rationalization functioning as a moderator in our model. As a result, we find that the interaction hypotheses involving the correlations of PSW*RNL and PRT*RNL with whistleblowing intention are fully supported. Specifically, we found that the relationships between PSW*RNL = whistleblowing intention, PTR*RNL = whistleblowing intention were significant, with beta (β) values of −0.3118, 95% CI = (−0.4768, −0.1469), and −0.1578, 95% CI = (−0.3063, −0.0094), respectively, and significance at *p* ≤ 0.05. From these results, we conclude that Hypothesis 2, and Hypotheis 4b are fully supported. The interactions between PSW and rationalization are represented in Figure 2 at a standard deviation from rationalization mean of +1/−1. By using a simple slope test, we were able to estimate the strength of the positive correlations between perceived seriousness of wrongdoing and whistleblowing intention at both high and low levels of rationalization. The simple slope test showed a significant positive relationship (β = 1.2708, *p* < 0.001) for employees with low rationalization, and a significant positive association (β = 0.7929, *p* < 0.001) for employees with high rationalization (see Table 6). Examine the moderating effect of RNL between perceived threat of retaliation and whistleblowing, Table 7 clarifies the moderating effect (β = −0.1578 ***), supporting Hypothesis 4a. In Figure 3, PTR*RNL interactions are depicted at +1/−1 standard deviation from rationalization mean. Slope tests were utilised to assess the positive associations between perceived threat of retaliation and whistleblowing intention at high and low rationalization levels. The simple slope test showed a significant positive relationship (β = 0.6421, *p* < 0.001) for employees with low rationalization, and a significant positive association (β = 0.4002, *p* < 0.001) for employees with high rationalization. Thus, hypothesis Hypotheis 4a is confirmed.

## 5. Conclusions and Discussion

Whistleblowing may be used as a preventative approach to reduce the likelihood of misconduct and discrepancies occurring. It is more likely that managers will identify inconsistencies early on if employees and other key stakeholders are given the authority to raise the alarm when anything is wrong. The implementation of steps to enable whistleblowing in companies that take their Code of Conduct seriously will include the establishment of a secure corporate whistleblowing system or hotline, and the development of policies and procedures for reporting misbehaviour.

The whistleblowing approach have been investigated and recognized as a mechanism for exposing misconduct in organizations by academics from a broad variety of fields. Employees from Pakistan’s telecom industry are used as a sample in this study, which is based on the theory of planned behavior. We examine the impacts of perceived seriousness of wrongdoing and perceived threat of retaliation on whistleblowing intention, as well as the mediating roles of anticipated regret and the moderating roles of rationalization [50]. In particular, the role of rationalization was investigated to determine whether people should blow the whistle based on perceived seriousness of wrongdoing and perceived threat of retaliation levels. Individuals’ motivations for reporting wrongdoing have been investigated in a number of studies; however, little is known about what drives people to go public with their concerns. Our findings answer the research calls of [19,20,47], and these preliminary findings collected in the Pakistani setting will help fill empirical gaps in the existing research. According to the findings of recent research, the most important hazards that employees feel are “pressure from peers” and “verbal harassment or intimidation,” respectively. Whistleblowing in the context of the most serious wrongdoing may sometimes result in “significant damage to the whistleblower”. This sample supports whistleblowing based on the possibility of ‘helping victims’, who are suffering as a consequence of wrongdoing, rather than on other factors. In conclusion, the internal reporting channel, which is defined as “reporting… to the relevant personnel inside the organization,” was identified as the route most often used by our respondents to report misconduct. These findings imply that whistleblowers are motivated to expose wrongdoing via their contacts and support from peers. Whistleblowers may opt to remain silent in the workplace because of the threat of retaliation. The whistleblower reports wrongdoings due to the negative and serious consequences faced by the organization or employees. Whistleblowing is made possible by supporting and backing the whistleblower in risky situations, ultimately protecting the organization and employees from harm and damage. In some scenarios, a link exists between the perceived seriousness of wrongdoing and perceived threat of retaliation, depending on the seriousness of the wrongdoings. When wrongdoing is serious, and the wrongdoer is powerful, there is retaliation from the other side. However, as reported by [18,36,48], whistleblowers face no threat of retaliation, but when they face retaliation, this negatively affects the whistleblower’s mental health. There are no specific laws enforced in Pakistan, and organizations implement their mechanisms in their respective organizations to prevent loss to organizations and employees. Usually, in organizations the whistleblower shows hesitation to report wrongdoings because of the lack of a proper mechanism in the organization or lack of interest from higher management in this regard [32].

Our hypothesis was put to the test, and we found five key results. First, we tested the link between perceived seriousness of wrongdoing and whistleblowing intentions. We found evidence of a positive relationship between perceived seriousness of wrongdoing and whistleblowing intention. Whistleblowers are more inclined to blow the whistle when they notice major wrongdoing in the workplace. Our results support data from prior research on the seriousness of wrongdoing [14,17,26,28,29]; the greater the potential for wrongdoing to cause damage, the more likely the observers are to report it. Furthermore, the seriousness of wrongdoing instills a feeling of personal responsibility to prevent possible damage to victims. As a result, the observer becomes more likely to speak out and take action. Second, perceived threat of retaliation seems to reduce people’s desire to come forward with knowledge regarding unlawful acts, as shown by a link between PTR and the intention of making a whistleblower report. In other words, the higher the perceived threat to whistleblowers, the more likely it is that they will opt to remain silent. Our results support earlier research on the prospect of retaliation [5,31,68,70]. When the perceived degree of retaliation is minor, whistleblowers may choose to use an internal or anonymous reporting route to bring attention to wrongdoing [10,31,57,59]. Companies should choose someone from inside the organization to listen to workers’ concerns about wrongdoing in this respect, as it seems that observers prefer to report wrongdoings to someone who has a neutral profile rather than to their superiors or upper-level management officials. Third, we found evidence that rationalization plays a crucial role in regulating the connection between perceived seriousness of wrongdoing and perceived threat of retaliation. We suggest that the rationalization process assists observers in persuading them, before whistleblowing, to be aware of retaliation: “(a) there is legal protection for whistleblowers, and therefore threats can be minimized; (b) they are able to report wrongdoings via anonymous channels, or (c) they will be supported by bystanders and superiors.” Consequently, the rationalization approach is likely to minimize the perception of threats as obstacles to whistleblowing [14,24]. This confirms earlier research that found a link between rationalization and the whistleblowing intention [21,22,94,95]. Fourth, we found evidence that rationalization plays a crucial role in regulating the connection between perceived seriousness of wrongdoing and whistleblowing intention [35]. The significance of rationalization’s involvement in this connection is amply substantiated by our findings. Using rationalization as a moderator, the whistleblower is better able to examine the probable repercussions and disadvantages of wrongdoing, as well as strengthening the sensation that wrongdoing has occurred. As a consequence, the rationalization mechanism has a tendency to increase the urge to raise the warning sign [21,24]. Finally, our findings confirm the hypothesis that anticipated regret mediates the connection between perceived seriousness of wrongdoing and whistleblowing intention [26,96]. The feeling of anticipated regret is founded on analysis of the situation. Deciding is difficult in uncertain times, and decision makers anticipate future regret when they learn their options are limited [97]. In addition, “when the most preferred alternative is not necessarily superior to another alternative” [98], they experience anticipated regret. Because people are regret-averse, they may choose a regret-avoiding alternative [97,99,100].

### 5.1. Implications for Theory

The findings of our study, which we discuss in more detail below, have a wide range of theoretical and practical implications. This study makes one of the most significant contributions to the literature on whistleblowing because it is the first to examine anticipated regret as a mediator in the relationship between perceived seriousness of wrongdoing and whistleblowing intention, and RNT as a moderator in the relationship between perceived seriousness of wrongdoing, perceived threat of retaliation, and whistleblowing intention. Despite earlier study focusing on the individual, situational, and contextual aspects that impact whistleblowing intention, the process of determining whether to blow the whistle still remains unclear [15,19,35]. Research in this area lends credence to our claim that the RNL has an impact on perceived seriousness of wrongdoing and perceived threat of retaliation, regarding the intent to report wrongdoing. Furthermore, our findings unify conflicting evidence on the association between perceived seriousness of wrongdoing, perceived threat of retaliation, and whistleblowing intention, pointing to the importance of a third element in moderating the relationship [35]. However, although previous research on this issue has shown conflicting results, our findings imply that rationalization may assist individuals in persuading themselves of the seriousness of wrongdoing and, as a result, in evoking the urge to report it [29,35]. The following are some of the practical ramifications of our findings: first and foremost, there is increased concern about wrongdoing in organisations, and it is critical to understand why people who see wrongdoing decide to come forward without considering threats [2].

### 5.2. Managerial Implication

We believe that managers and other stakeholders in organisations will be able to use our results to reduce the potential of retribution against whistleblowers, while also assisting them by offering freely available reporting channels to the general public [14]. Increased trust in the ability of observers to report wrongdoings should be facilitated by the use of internal channels. Observers prefer to report wrongdoings to someone who has an impartial profile, rather than to their superiors or upper-level management, according to the findings of this research. In this case, the senior management of companies should identify individuals inside the company who have the potential to play the role of mediator. Internal channels for dealing with disclosures must also be established, and they must have specific mechanisms in place to safeguard those who come forward with information. Second, management must adopt programs such as incentives for whistleblowers when they expose misconduct in order to minimize additional damage and enhance internal controls when employees are active participants in the observation of wrongdoing inside an organization. A successful whistleblowing system must contain clearly defined lines of authority, reporting methods, and training on fraud education in the workplace to aid employees in spotting serious wrongdoing in their organizations early in the process. This will contribute to improving the overall effectiveness of the system [13].

### 5.3. Limitations and Future Research Directions

Certain limitations apply to this study. Our research does not take into account elements that might alter the association between relevant factors, such as demographics [101]. For example [6], when it comes to blowing the whistle, women are more likely than males to face retaliation. In addition, [6] report that, compared to men, women are more likely to bring serious wrongdoing to the notice of the authorities. More importantly, relying on surveys to gauge respondents’ emotions in the face of a hypothetical whistleblowing scenario has apparent drawbacks, since the answers supplied may differ from those experienced in real-life circumstances. Our main results, on the other hand, may or may not be relevant in different cultural situations. Several scholars have shown that cultural settings, such as the gap between collectivism and individualism, have a significant impact on the chance that someone would report an illegal behaviour when it occurs [102,103]. A study of whistleblowing intention across cultures offers early data to substantiate the influence of cultural variations on whistleblowing intention [104]. Final thoughts: our results only provide credence for a realistic approach to individuals making whistle-blowing decisions. When it comes to the aim of whistleblowers and ethical decision-making, a non-rationalist perspective has recently been taken into consideration [105]. When considering whether or not to blow the whistle, it is crucial to weigh up emotions and instincts, as well as rationale. For further study, we recommend the following possibilities. First, study is required to look at mediators and moderators that may help us better understand the processes that control whistleblowing in organisational settings and provide new areas of opportunity for changing organisational policies. Our study research revealed that anticipated regret serves as a mediator between PSW and whistleblower intentions. It is proposed by [106] that anger can play a mediating role in whistleblowing incidents, and that other emotions (such as guilt and shame) may also play a mediating function, as mentioned by [76]. More research is needed to determine if certain personality characteristics (e.g., perspective taking) have a significant moderating influence on this situation. It is recommended that future research examine cross-cultural comparisons of the factors that influence whistleblowers’ intentions [21,105].

## Figures and Tables

**Table 1:** Sample Characteristics.

Demographic Variables	Frequency	Percentage (%)
Gender		
Male	221	62.78
Female	133	37.21
Age (yrs)		
25–29	24	6.8
30–34	204	57.6
35–39	69	19.5
Over 40	57	16.1
Tenure (yrs)		
1–7	269	76
8–15	60	16.9
16–25	12	3.4
Over 25	13	3.7
Qualifications		
HSSC	17	4.8
Bachleor	65	18.5
Master	231	65.3
MS/PhD	41	11.6

Note: HSSC (Higher Secondary School Certificate).

**Table 2:** Results of confirmatory factor analysis (N = 354).

Model	χ^2^/df	RMR	GFI	CFI	RMSEA
Baseline model (Five-factor model) ^o^	2.03	0.03	0.89	0.91	0.03
4-factor model ^a^	4.47	0.07	0.92	0.93	0.08
3-factor model ^b^	2.67	0.06	0.91	0.90	0.08
2-factor model ^c^	1.72	0.02	0.94	0.91	0.07
1-factor model ^d^	5.57	1.04	0.47	0.37	0.18

Note: ^a^ Combining PSW & Anticipated Regret, ^b^ Combining PSW, Anticipated Regret & Whistleblowing, ^c^ Combining PTR, Anticipated Regret & RNL ^d^ PSW, PTR & Anticipated Regret, RNL, whistleblowing intention, ^o^ combining all items. PSW: Perceived serious of wrongdoing, PTR: Perceived threat of retaliation, RNL: Rationalization, RMSEA: Root mean square error of approximation, RMR: The root mean square residual, CFI: The comparative fit index, GFI: The Goodness-of Fit Index, χ^2^/df: Chi-square divided by the degrees of freedom.

**Table 3:** Mean, Standard Deviation, Correlations and Reliability (N = 354).

Variables	Mean	SD	CR	AVE	VIF	1	2	3	4	5	6	7	8	9
1.Gender	1.3814	0.49220	-	-										
2.Age	2.4492	0.84086	-	-		−0.011								
3.Tenure	2.7994	0.54994	-	-		−0.020	−0.019							
4.Qualification	2.0282	0.70151	-	-		−0.204 **	0.017	0.059						
5.WBI	3.8164	0.72057	0.91	0.57	1.231	0.123 *	0.013	−0.003	−0.055	(0.77)				
6.PSW	3.7238	0.51168	0.89	0.53	1.312	0.075	−0.018	−0.063	−0.061	0.757 **	(0.87)			
7.PTR	3.5706	0.66464	0.92	0.54	1.142	0.033	−0.004	0.072	0.036	0.470 **	0.536 **	(0.81)		
8.RNL	1.9944	1.25275	0.91	0.52	1.156	−0.006	−0.008	−0.007	0.027	0.022	0.061	0.211 **	(0.84)	
9.AR	2.4076	0.99651	0.89	0.56	1.265	0.006	0.007	−0.008	0.052	0.018	0.113 *	0.287 **	.877 **	(0.88)

Note: *. Correlation is significant at the 0.05 level (2-tailed). **. Correlation is significant at the 0.01 level (2-tailed). WBI = Whistleblowing Intention, PSW = Perceived Seriousness of Wrongdoing, PTR = Perceived Threat of Retaliation, AR = Anticipated Regret, RNL = Rationalization. CR: Composite Reliability, AVE: Average variance extracted, VIF: Variance inflation factor

**Table 4:** Results of hierarchical regression analyses (N = 354).

	Anticipated Regret		Whistleblowing Intention		
	Model 1	Model 2	Model 3	Model 4	Model 5
Control Variables					
Age	0.009	0.011	0.013	0.025	0.014
Gender	0.013	−0.004	0.172	0.100	0.143
Education	0.080	0.088	0.002	0.061	−0.041
Tenure	−0.020	−0.002	−0.032	0.002	−0.052
Independent Varibles					
PSW	0.220 **		10.066 **		
PTR				−0.509 **	
Mediator					
Anticipated Regret					0.013 *
Moderator					
RNL					
Interaction					
PSW*AR					−0.11 **
PRT*AR					0.082 **
ΔF	0.302	0.258	0.561	0.960	0.293
R^2^	0.003	0.017	0.016	0.580	0.236
ΔR^2^	0.003	0.004			

N = 354; ** *p* < 0.01; * *p* < 0.05 (Two-tailed test). PSW: Perceived serious of wrongdoing, PTR: Perceived threat of retaliation, AR: Anticipated regret, ΔF: Change in F distribution, R^2^: the proportion of variance in the dependent variable, ΔR^2^: Change in the proportion of variance in the dependent variable.

**Table 5:** Mediation Analysis.

	t	95%CI		SE	β
		LL	UL		
Step 1					
PSW → Anticipated Regret	2.13	0.005	0.112	0.509	0.058 **
Anticipated Regret → Whistleblowing Intention	15.30	0.537	0.696	0.041	0.013 *
	BootLLCI	BootULCI	Boot SE	β	Decision
Step 2					
Mediation Path	−0.0404	−0.0016	0.0099	0.0168	Partial Mediation

Note: *. Correlation is significant at the 0.05 level (2-tailed). **. Correlation is significant at the 0.01 level (2-tailed). t = the size of the difference relative to the variation in your sample data, SE: Standard Error, β: The beta coefficient, BootLLCI: Lower Limit confidence interval, BootULCI: Uper limit confidence interval.

**Table 6:** Moderation Analysis.

RNL(W)					
Variables	PSW (X)			Whistleblowing (Y)	
	t	95%CI		SE	β
		**LL**	**UL**		
PSWxRNL	−3.7181	−0.4768	−0.1469	0.0839	−0.3118 *
RNL (−1 SD)	1.2062	1.1278	1.4138	0.0727	1.2708 ***
RNL (+1 SD)	2.7385	0.6182	0.9677	0.0888	0.7929 ***

RNL(W): Rationalization as a moderator, PSW(X): Perceived seriouness of wrongdoings as a independent variable, PTR(X): Perceived Treat of Retaliation as independent variable. PSWxRNL: Interaction of Perceived seriouness of Wrongdoings and Rationalization. RNL(−1SD): Rationalization at −1 Standard Deviation, RNL(+1SD): Rationalization at +1 Standard Deviation, LL: Lower Limit, UL: Upper Limit, SE: Standard Error, β: Confidence interval. *. Correlation is significant at the 0.01 level (2-tailed). ***. Correlation is significant at the 0.001 level (2-tailed).

**Table 7:** Moderation Analysis.

RNL(W)					
Variables	PTR (X)			Whistleblowing (Y)	
	t	95%CI		SE	β
		**LL**	**UL**		
PTRxRNL	−2.0913	−0.3063	−0.0094	0.0755	−0.1578 *
RNL (−1 SD)	8.9086	0.5003	0.7838	0.0721	0.6421 ***
RNL (+1 SD)	4.6647	0.2315	0.5689	0.0858	0.4002 ***

RNL(W): Rationalization as a moderator, PSW(X): Perceived seriouness of wrongdoings as a independent variable, PTR(X): Perceived Treat of Retaliation as independent variable. PSWxRNL: Interaction of Perceived seriouness of Wrongdoings and Rationalization. RNL(−1SD): Rationalization at −1 Standard Deviation, RNL(+1SD): Rationalization at +1 Standard Deviation, LL: Lower Limit, UL: Upper Limit, SE: Standard Error, β: Confidence interval. *. Correlation is significant at the 0.01 level (2-tailed). ***. Correlation is significant at the 0.001 level (2-tailed).

## Data Availability

By request to the authors of correspondence.
